# Memory T Cell Subpopulations as Early Predictors of Remission to Vedolizumab in Ulcerative Colitis

**DOI:** 10.3389/fmed.2022.837294

**Published:** 2022-06-15

**Authors:** Maria Gonzalez-Vivo, Minna K. Lund Tiirikainen, Montserrat Andreu, Agnes Fernandez-Clotet, Alicia López-García, Francisca Murciano Gonzalo, Lourdes Abril Rodriguez, Carmen de Jesús-Gil, Ester Ruiz-Romeu, Lídia Sans-de San Nicolàs, Lluis F. Santamaria-Babí, Lucía Márquez-Mosquera

**Affiliations:** ^1^Department of Gastroenterology, Hospital del Mar, Barcelona, Spain; ^2^IMIM (Hospital del Mar Medical Research Institute), Barcelona, Spain; ^3^Grup d’Immunologia Translacional, Departament de Biologia Cel⋅lular, Fisiologia i Immunologia, Facultat de Biologia, Universitat de Barcelona (UB), Parc Científic de Barcelona (PCB), Barcelona, Spain; ^4^Department of Gastroenterology, Hospital Clinic de Barcelona, Barcelona, Spain

**Keywords:** inflammatory bowel disease, ulcerative colitis, biological therapy, integrins, T lymphocytes

## Abstract

**Background:**

Vedolizumab is a humanized monoclonal antibody targeting the α_4_β_7_ integrin used for the treatment of ulcerative colitis. Few biomarkers related to vedolizumab response have been identified. The aim of this work was to assess whether baseline circulating CD4^+^ and CD8^+^ memory T-lymphocyte subpopulations could help to identify patients with response to vedolizumab treatment in ulcerative colitis.

**Methods:**

Prospective pilot study in 15 patients with active ulcerative colitis and previous failure to anti-TNFα starting vedolizumab treatment. Peripheral blood samples were obtained before the first dose of vedolizumab and at week 6 and 14 of treatment. Clinical remission was defined as a Mayo Clinic partial score of ≤2 points without any concomitant dose of steroids. Biochemical remission or endoscopic improvement was defined as fecal calprotectin <250 mcg/g or Mayo endoscopic subscore ≤1.

**Results:**

At week 14, nine patients achieved clinical remission and eight patients achieved biochemical remission or endoscopic improvement. Patients in clinical remission presented higher baseline CD8 α_4_β_7_^+^ memory T cells concentration when compared with patients with no remission. In addition, patients with biochemical remission or endoscopic improvement at week 14 presented higher baseline concentration of CD8 α_4_β_7_^+^ memory T cells. No differences were identified according to flare severity, extent of disease or type of anti-TNFα failure. There were no significant differences regarding changes in T cell subsets during vedolizumab induction.

**Conclusion:**

CD8^+^ α_4_β_7_^+^ memory T cells before starting vedolizumab therapy could be an early predictor of remission in ulcerative colitis patients and therefore help to select a subset of responders.

## Introduction

Ulcerative colitis (UC) is a chronic, relapsing, inflammatory disorder of the gastrointestinal tract affecting an increasing number of individuals in industrialized countries (1, 2). It is a subtype of inflammatory bowel disease (IBD), which also includes Crohn’s disease (CD).

Treatment of UC includes salicylates, systemic corticosteroids, immunomodulators, and monoclonal antibodies (3). Treatment should be tailored to disease activity (mild, moderate, severe), extent and phenotype (4–6).

Vedolizumab (VDZ) is a humanized monoclonal antibody directed against gut-homing integrin α_4_β_7_. It prevents T lymphocyte adhesion to the vascular endothelium [*mucosal addressing cell adhesion molecule 1* (MAdCAM-1) and fibronectin], expressed in the intestinal tract (7). VDZ has demonstrated a therapeutic effect in UC and CD (8, 9).

The administration of VDZ is followed by a significant expansion of α_4_β_7_^+^ memory helper T lymphocytes in peripheral blood while their frequency in gastrointestinal tissues decreases in primates (10). In humans, VDZ induces qualitative and quantitative changes in a subset of memory T cells (11) as well as several effects on innate immunity (changes in macrophage populations, pronounced alterations in the expression of molecules involved in microbial sensing, chemoattraction and regulation of the innate effector response) (12). Hence, studying the changes in circulating memory T cells in UC patients treated with VDZ could lead to identify molecular predictors of response to this treatment. Although recently some clinical and biochemical predictive factors of VDZ response in IBD have been described (13–16), data on molecular markers are still scarce in UC (17, 18). In this scenario, identifying biomarkers of response to VDZ in UC would be a useful tool to select a subset of patients who would be likely to respond to VDZ, rather than follow the current sequential treatment failure approach.

Therefore, the aim of this study was to assess whether baseline circulating CD4^+^ and CD8^+^ α4β7^+^ memory T cell subpopulations, several lymphocytic markers previously involved in the physiopathology of IBD (19, 20), and their changes during treatment could be predictors of response to VDZ in patients with UC.

## Methods

### Study Population

We conducted a prospective observational study including UC patients recruited consecutively at the Hospital del Mar IBD Unit from January 2017 to June 2018. All patients were diagnosed of UC following ECCO criteria (21) and they received VDZ treatment in a standard induction plan (300 mg i.v. 0–2–6 weeks). Patients who were in clinical response at week 14 received VDZ 300 mg i.v. every 8 weeks as maintenance therapy. The washout period for previous anti-TNFα treatment was established per protocol as 4 weeks for infliximab i.v. and 2 weeks for adalimumab s.c. During the induction period, oral systemic corticosteroids and oral prolonged steroids (beclomethasone dipropionate) were allowed meanwhile any other immunosuppressant therapy were forbidden.

Before starting VDZ, disease activity was evaluated using the Mayo clinical score, including endoscopic activity confirmed by colonoscopy (Mayo endoscopic subscore 2 or 3). Bacterial and parasitic infections were ruled out by stool culture and cytomegalovirus was excluded in colonic biopsies by immunohistochemistry.

### Data Collection

Peripheral blood samples were collected from patients prior to starting VDZ treatment and at week 6 and 14, immediately before VDZ administration. Stool sample collection was performed 1–3 days before starting VDZ treatment and before week 14. All stool samples were analyzed to measure fecal calprotectin (FC) by an automated immunoassay (Phadia EliA*™* Calprotectin; normal range from 0 to 50 mcg/g). In addition, the partial Mayo score was prospectively calculated at weeks 6 and 14. Demographic and clinical data including age, gender, disease duration, disease extent, concomitant medications, endoscopic activity, histology, albumin, and serological inflammatory marker levels were collected from medical records.

The primary endpoint was to evaluate whether baseline circulating CD4^+^/CD8^+^ α_4_β_7_^±^ memory T cells as well as several surface markers (HLA-DR, CCR9), Th17 phenotype marker IL23R and intracellular IL17A and IL9, predict clinical remission to VDZ at week 14.

The secondary end-points were:

- To assess whether the subsets of memory T cells (α_4_β_7_, HLA-DR, IL23R, CCR9, IL17A, IL9, β_7_, and β_7_-CCR9) at baseline predict endoscopic and biochemical remission at week 14, and sustained clinical remission at week 52.
- To assess whether changes in the same memory T cell subsets during VDZ treatment are related to clinical and biochemical remission or endoscopic improvement.

### Definitions of Response

Clinical response was defined as a decrease in the partial Mayo Clinic score of at least three points at week 14. Clinical remission was defined as a Mayo Clinic partial score of ≤2 points without any concomitant dose of steroids at week 14. Sustained clinical remission was defined as a Mayo Clinical partial score of ≤2 points without concomitant corticosteroid therapy at week 52. Biochemical remission was defined as FC < 250 mcg/g, as considered in GETECCU Spanish guidelines (22, 23). Endoscopic improvement was defined as a Mayo endoscopic subscore ≤1 (24, 25).

### Circulating Memory T Cell Isolation

Peripheral blood mononuclear cells (PBMC) were isolated by Ficoll gradient (GE Healthcare, Princeton, NJ, United States) and SepMate PBMC isolation tubes (STEMCELL Technologies, Grenoble, France). Then, memory T cells were purified after two sequential immunomagnetic separations consisting of the CD14^+^/CD19^+^ and CD45RA^+^/CD16^+^ cell depletions (Miltenyi Biotec, Bergisch Gladbach, Germany). Purified circulating memory T cells were cryopreserved in aliquots in liquid nitrogen using established techniques.

### Circulating Memory T Cell Populations Staining and Flow Cytometry Analysis

One day prior staining, circulating memory T cells were thawed and plated at 1M cells/ml in RPMI (Sigma-Aldrich, St. Louis, MO. United States) with 10% fetal bovine serum (Gibco, Grand Island, NY, United States) and 1% penicillin-streptomycin (Sigma-Aldrich), after cell viability assessment. Next day, cells were plated at 2M cells/ml in the presence of Brefeldin A solution (1 μl Brefeldin per 500 μl of final volume) (BioLegend, San Diego, CA, United States) and incubated at 37°C for 4 h.

The following antibodies were used for the multicolor flow cytometry staining: CD4-PE Texas Red (Life Technologies, Carlsbad, CA, United States), CD8-AF700 (BioLegend) α_4_ integrin (CD49d)-BV510 (BioLegend), β_7_ integrin-FITC (Affymetrix, eBioscience Inc., Santa Clara, CA, United States), HLA-DR-ACP/Cy7 (BioLegend), IL23R-PE (R&D Systems, United States), CCR9-PerCP-Cy5.5 (BioLegend), and intracellular IL17A-BV421 (BioLegend) and IL9-APC (Miltenyi Biotec). FOXP3 Fix/Perm Buffer Set (BioLegend) for the intracellular staining was used. Samples were resuspended in 400 μl sheath cytometer buffer and 100 μl AccuCheck Counting Beads (Invitrogen, Carlsbad, CA, United States) were added for the absolute cell subset counts.

Circulating memory T cell subsets were acquired through the Beckman Coulter Gallios Flow Cytometer at the core facility of the Parc Científic de Barcelona and FlowJo software was used for analysis gating using respective isotype control antibodies and adequate flow cytometer compensations.

Finally, we identified in peripheral blood the following memory T cell subpopulations: α_4_β_7_, HLA-DR, IL23R, CCR9, IL17A, IL9, β_7_, and β_7_-CCR9.

### Statistical Analysis

The study was designed as a proof of concept and standard sample size could not be calculated due to the absence of published previous data.

Dichotomous variables were presented as percentages, and *p-*value associations were determined with χ^2^ or Fisher exact tests. For continuous variables, data were presented as median and interquartile range (IQR). Normally distributed data were analyzed by unpaired sample *t-*test. Abnormally distributed data were compared by non-parametrical tests (Mann–Whitney *U* test). Wilcoxon signed-rank test was used to compare immune subsets before and after VDZ therapy.

All *t-*tests were two-sided and *p*-values < 0.05 were considered statistically significant. No adjustments for multiple comparisons were performed, as this was a hypothesis-generating study and many of the outcomes measured were biologically related. Statistical analyses were performed using SPSS 25.0 software (Statistical Package for the Social Sciences Inc., Chicago, IL, United States).

### Ethical Considerations

All study subjects provided written informed consent before enrollment. Research procedures were approved by the Hospital del Mar Clinical Research and Ethics Committee in 2016. This study were conducted according to the principles expressed in the 1975 Declaration of Helsinki (6th revision, 2008) in the Council of Europe Convention on Human Rights and Biomedicine.

## Results

### Patients Characteristics

A total of fifteen UC patients starting VDZ treatment were included prospectively, seven with severe disease (partial Mayo score between 7 and 9 points).

All patients had received anti-TNFα previously: nine patients were primary non-responders and six patients presented loss of response.

Steroids were started at a standard dose simultaneously with VDZ: prednisone 1 mg/kg/day in 12 patients and beclomethasone dipropionate 5 mg/day in 1 patient. Two patients did not take any concomitant treatment. Steroids were tapered and completely discontinued between week 6 and 10.

After induction, 11 patients provided stool samples to measure to measure FC and 12 patients underwent and 12 patients underwent a colonoscopy.

At week 14, nine patients achieved clinical remission, five patients were in biochemical remission, six patients presented endoscopic improvement and eight patients achieved biochemical remission or endoscopic improvement.

At week 52, ten patients were in sustained clinical remission: nine received VDZ every 8 weeks and one patient received VDZ every 8 weeks, oral mesalazine and tacrolimus.

Clinical and demographic characteristics of patients depend on clinical remission are shown in Table 1.

**TABLE 1:** Comparison of demographic and clinical characteristics between patients achieving or not clinical remission at week 14.

	Remitters (*n* = 9)	Non-remitters (*n* = 6)	*p-*value
***Female sex, No. (%)***	5 (55.6)	2 (33.3)	0.302
***Age (years)- median (IQR)***	45 (31.5–66)	41.5 (33.5–52.8)	0.750
***Disease extent, No. (%)***			0.411
*E1 – Proctitis*	1 (11.1)	0	
*E2 – Left sided colitis*	4 (44.4)	4 (66.7)	
*E3 – Pancolitis*	4 (44.4)	2 (33.3)	
***Severity, No. (%)***			
*Severe (Mayo score 7–9)*	4 (44.4)	3 (50)	0.696
***Disease duration, No. (%)***			0.441
<*10 years*	7 (77.8)	1 (16.7)	
*Between 10 and 20 years*	1 (11.1)	5 (83.3)	
>*20 years*	1 (11.1)	0	
***TNF antagonist failure, No. (%)***			0.418
*Primary non-responders*	7 (77.8)	2 (33.3)	
*Loss of response*	2 (22.2)	4 (66.7)	
***Co-treatment at baseline, No. (%)***			0.036^a^
*Oral prednisone*	9 (100)	3 (50)	
*Oral beclomethasone*	0	1 (16.7)	
*None*	0	2 (33.3)	
***Albumin (g/dl), median (IQR)***	4.2 (3.9–4.6)	4 (3.5–4.6)	0.331
***C Reactive Protein (mg/dl), median (IQR)***	0.51 (0.1–1.54)	0.56 (0.13–2.02)	0.801
***Fecal Calprotectin (mcg/g), median (IQR)***	619 (134–1767)	849 (311–3878)	0.308

*^a^p-value < 0.05.*

### Memory T Cell Subpopulations Before Treatment (at Baseline)

The concentration of the different CD4^+^ and CD8^+^ memory T cell subpopulations were studied in our cohort of UC patients before treatment. Results of one patient at baseline were excluded from the final statistic analysis due to technical problems with blood samples that led to massive cell death.

**FIGURE 1:**
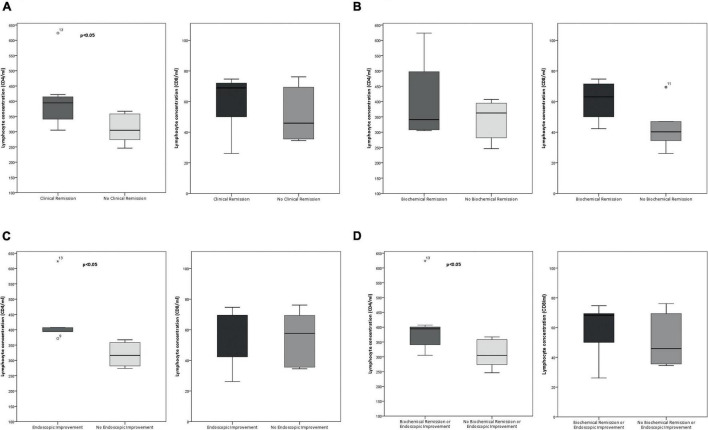
Boxplots of total CD4^+^ (left) and CD8^+^ (right) lymphocytes concentration at baseline depend on each type of remission (values are shown in cells per milliliter). **(A)** Clinical remission at week 14. **(B)** Biochemical remission. **(C)** Endoscopic improvement. **(D)** Biochemical remission or endoscopic improvement. Outliers are shown as circles and extreme outliers, as *.

**FIGURE 2:**
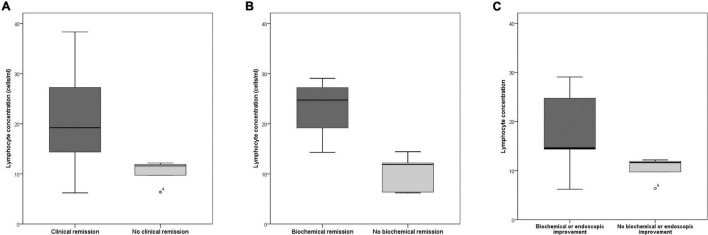
Boxplots of CD8 α_4_β_7_^+^ memory T cells concentration at baseline depend on each type of remission (values are shown in cells per milliliter). All differences are statistically significant. **(A)** Clinical remission at week 14. **(B)** Biochemical remission. **(C)** Biochemical or endoscopic improvement. Outliers are shown as circles.

**FIGURE 3:**
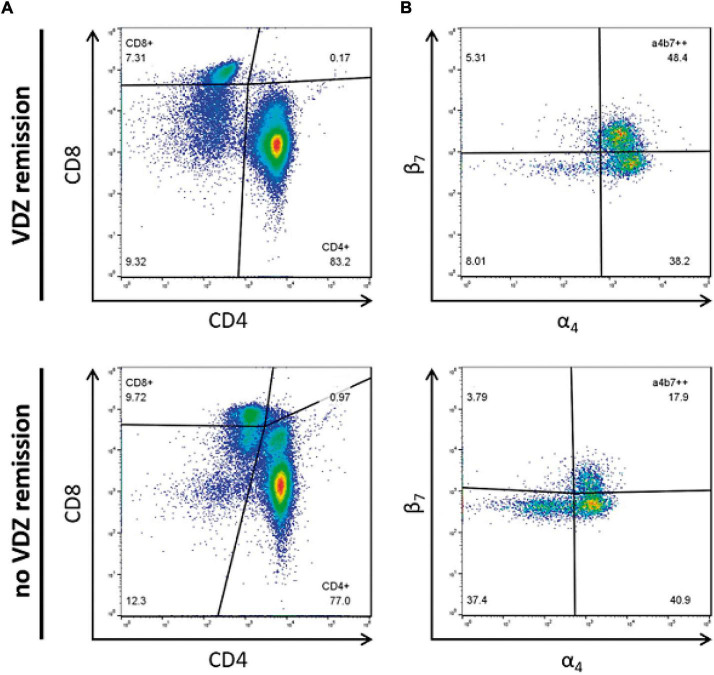
Representative flow cytometric analysis of baseline memory T cell subsets. Differential expression of **(A)** total CD4 versus CD8 memory T cells and **(B)** CD8 α_*4*_β_*7*_^+^ memory T cell subset. Values are shown as percentages.

Patients who achieved clinical remission at week 14, presented significantly higher CD4^+^ memory T cells and CD8^+^ α_4_β_7_^+^ memory T cells concentration compared with those who were not in clinical remission [median: 394.47 cells/ml versus 304.73 cells/ml, *p* = 0.02 (Figure 1A); 19.27 cells/ml versus 11.63 cells/ml, *p* = 0.02 (Figure 2A), respectively]. No significant differences were found in CD4^+^ memory T cells subsets between both groups. A representative flow cytometry plot is shown in Figure 3.

Patients who were in sustained clinical remission at week 52 presented higher CD4^+^ memory T cells and CD8 α_4_β_7_^+^ memory T cells concentration compared with non-remitters (median: 394.47 cells/ml versus 327.66 cells/ml, *p* = 0.02; 14.43 cells/ml versus 11.85 cells/ml, *p* = 0.02, respectively).

The CD8^+^ α_4_β_7_^+^ memory T cells concentration in patients with biochemical remission was significantly higher (median: 24.75 cells/ml versus 11.87 cells/ml, *p* = 0.019) than in patients who did not achieved biochemical remission (Figure 2B). The CD8^+^ CCR9^+^ memory T cells concentration was significantly lower in biochemical remitters than in non-remitters (median: 0.29 cells/ml versus 1.12 cells/ml, *p* = 0.019). There were no statistically significant differences in CD4^+^ memory T cell subsets between both groups (Figure 1B).

Regarding endoscopic improvement, the CD8^+^ α_4_β_7_^+^ memory T cells concentration was higher in patients with endoscopic improvement than in patients who did not show endoscopic improvement, but these differences did not reach statistical significance (median: 14.43 cells/ml versus 11.63 cells/ml, *p* = 0.43). CD4^+^ memory T cells concentration was significantly higher in patients with endoscopic improvement compared with those without endoscopic improvement (median: 394.47 cells/ml versus 316.38 cells/ml, *p* = 0.004) (Figure 1C). Again, no statistically differences in CD4^+^ memory T cell subsets were identified between both groups.

Finally, patients who were in biochemical remission or presented endoscopic improvement had significantly higher CD4^+^ and CD8^+^ α_4_β_7_^+^ memory T cells concentration compared with those without biochemical remission or endoscopic improvement [median: 394.47 cells/ml versus 304.73 cells/ml, *p* = 0.02 (Figure 1D); 14.43 cells/ml versus 11.63 cells/ml, *p* = 0.02 (Figure 2C), respectively].

In all CD4^+^ and CD8^+^ T cell subsets analyzed, no significant differences were identified according to flare severity, extent of disease or type of previous anti-TNFα failure.

Comparison between median of CD4^+^ and CD8^+^ T cell subsets depend on each type of remission is shown by bar graph in Supplementary Figure 1.

### Memory T Cell Subpopulations at Weeks 6 and 14

Regarding clinical and biochemical remission, there were no statistically significant differences in all CD4^+^ and CD8^+^ T cell subsets at week 6. Likewise, no significant differences were identified in the same T cell subpopulations at week 6 between patients who presented endoscopic improvement and those without endoscopic improvement (Supplementary Figure 2).

In relation to T cell subpopulations at week 14, results of two patients were excluded from the final statistic analysis due to technical problems with blood samples that led to massive cell death. CD8 β_7_^+^ memory T cell concentration was significantly higher in the group of patients that achieved clinical remission and biochemical remission or endoscopic improvement, compared with those who did not present any type of remission (median: 21.10 cells/ml versus 7.07 cells/ml, *p* = 0.03) (Supplementary Figure 3).

### Memory T Cell Subpopulations During Vedolizumab Treatment Induction Phase

During VDZ induction, since baseline until weeks 6 and 14, no statistically significant changes were observed in CD4^+^ and CD8^+^ memory T cell subsets concentration between patients presenting clinical remission and endoscopic improvement and patients who did not achieved remission.

## Discussion

In this prospective study, we investigated if several memory T cell subpopulations in peripheral blood could predict VDZ response in UC. A higher concentration of baseline CD8^+^ α_4_β_7_^+^ memory T cells was positively associated with clinical remission, biochemical remission or endoscopic improvement in UC patients after VDZ induction. This association was not related to flare severity, extent of the disease or type of anti-TNFα failure. In addition, a higher total CD4^+^ T cells concentration was also associated with clinical remission, biochemical remission or endoscopic improvement, although no statistically significant differences in CD4^+^ T cell subsets were identified between remitters and non-remitters.

Different studies have explored the role of lymphocyte subpopulations in the response to VDZ. According to our results, Boden et al. demonstrated -in 26 IBD patients- an increased α_4_β_7_^+^ expression in IBD responders to VDZ in multiple subsets of T, B, and NK cells, with terminal effector memory T cells (CD4 and CD8) and NK cells best discriminating between responders and non-responders (17). Apart of pretreatment α_4_β_7_^+^ expression, they found that α4β7 receptor saturation during maintenance therapy could be a candidate biomarker for vedolizumab response.

Otherwise, Fuchs et al. (26) analyzed retrospectively integrins and chemokine receptors on T cells before and during VDZ treatment in 17 UC and 19 CD patients. They found that increasing α_4_β_7_ levels in CD4^+^ T cells during induction period in UC were associated with favorable clinical response. Although patients with clinical response at week 16 had lower pretreatment frequencies of α_4_β_7_-expressing CD4^+^ T cells, these results included CD and UC patients, and, as no specific alterations of α4β7 integrin expression were founded in CD in this study, UC and CD patients should be analyzed separately.

Furthermore, a Belgian group published recently results from a prospective study in 71 IBD patients focused on baseline T cell subsets (27). Unlike our results, they observed in the UC cohort differences in the baseline proportion of CD4^+^ α_4_β_7_^+^ T cells between responders and non-responders, but not in the baseline proportion of CD8^+^ α_4_β_7_^+^ T cells. Despite the differences between T cells subsets, results could not be compared directly given both studies had different endpoints -clinical remission in our study and clinical response in the Belgian group-.

Besides, some studies focused on B cells or soluble proteins also supported the role of α_4_β_7_ as a predictor of response to VDZ. Uzzan et al. presented at the AGA Congress in 2018 a prospective study in 38 IBD patients (31 with UC) where a higher expression of pre-VDZ treatment α_4_β_7_^+^ on B cells predicted clinical remission at week 14 (28). Furthermore, a prospective study in 32 UC patients showed that patients who achieved clinical remission, soluble α_4_β_7_^+^ increased, whereas soluble MAdCAM-1, VCAM-1, ICAM-1, and TNF levels decreased rapidly (29).

Even though several groups have explored blood biomarkers, mucosal biomarkers had been broadly explored as predictors of response to VDZ treatment. Veny et al. analyzed the effect of VDZ treatment in the proportion of lymphocyte subsets and integrin expression both in colon biopsies and in blood samples (30). They included patients starting VDZ (*n* = 33), anti-TNFα (*n* = 45) and controls (*n* = 22). VDZ therapy specifically decreased α_4_β_*7*_^+^ CD4^+^ and CD8^+^ T cells in the colon, while preserving the proportion of α_*4*_β_*7*_^+^ plasma cells. However, this study was designed to understand the mechanism of action of VDZ and was not addressed to establish the association between baseline lymphocyte subpopulations and response to treatment.

Although mucosal biomarkers seemed very promising, we decided to investigate T cell subsets in peripheral blood as obtaining blood samples is minimally invasive for the patients and it can be easily applied in clinical routine. In addition, circulating CD8^+^ memory T cells are starting to attract attention in UC since they are activated in periphery (31) and present a clonal expansion in colon mucosa (32–34), which supports the relevance of our results for colon homing CD8^+^ T cells.

Some study limitations should be taken into account when interpreting our results: small sample size, as it was designed as an exploratory study, single-center cohort and differences in steroids treatment between groups. Therefore, additional studies will be needed to further validate our results in an independent and larger cohort and in order to elucidate if these results are associated exclusively with VDZ therapy.

Although it has also some strengths. It is a prospective study including a homogeneous and well-characterized cohort of UC patients with previous failure to anti-TNFα. The main goal, clinical remission at week 14, combined with an objective measurement of response (endoscopic improvement or calprotectin levels), was selected as a “real-life” endpoint. Likewise, T cell subpopulations were evaluated in peripheral blood as blood samples are routinely obtained in daily practice, which makes it easily reproducible.

In conclusion, in UC patients treated with VDZ, we have shown an association between high baseline CD8^+^ α_4_β_7_^+^, CD4^+^ T cells and clinical remission at week 14. Moreover, both are related to biochemical remission or endoscopic improvement. As a more specific subpopulation, assessing CD8^+^ α_4_β_7_^+^ T cell subset in peripheral blood might be a predictor of response that would help to support therapeutic decisions in routine clinical practice.

## Data Availability Statement

The raw data supporting the conclusions of this article will be made available by the authors, without undue reservation.

## Ethics Statement

The studies involving human participants were reviewed and approved by the Hospital del Mar Clinical Research and Ethics Committee. The patients/participants provided their written informed consent to participate in this study.

## Author Contributions

MA, LS-B, and LM-M contributed to the design of the study. MA, AF-C, AL-G, FM, LA, and LM-M included patients. MKLT, CJ-G, ER-R, and LS-dS analyzed the T cell subpopulations. MG-V, AF-C, MKLT, CJ-G, ER-R, and LS-dS collected the data. MG-V and LM-M analyzed the data. MG-V, LS-dS, and LM-M drafted the manuscript. CJ-G and LS-B critically revised the manuscript for important intellectual content. All authors read and approved the final manuscript.

## Conflict of Interest

The study funding was part of the Investigator Initiated Sponsored Research Program by Takeda. Design, recruitment, data analysis, and manuscript were performed independently by researchers at Hospital del Mar. Takeda Pharmaceuticals and associated employees did not intervene in any part of the process and did not have access to any of the data. The authors declare that the research was conducted in the absence of any commercial or financial relationships that could be construed as a potential conflict of interest.

## Publisher’s Note

All claims expressed in this article are solely those of the authors and do not necessarily represent those of their affiliated organizations, or those of the publisher, the editors and the reviewers. Any product that may be evaluated in this article, or claim that may be made by its manufacturer, is not guaranteed or endorsed by the publisher.

## Supplementary Material

Supplementary Figure 1 Median of CD4^+^ (left) and CD8^+^ (right) lymphocyte subpopulations concentration at baseline (values are shown in cells per milliliter). **(A)** Clinical remission. **(B)** Biochemical remission. **(C)** Endoscopic improvement. **(D)** Biochemical remission or endoscopic improvement.

Supplementary Figure 2 Median of CD4^+^ (left) and CD8^+^ (right) lymphocyte subpopulations concentration at week 6 (values are shown in cells per milliliter) depend on clinical remission. Similar results were found between patients with biochemical or endoscopic improvement and patients without improvement.

Supplementary Figure 3 Median of CD4^+^ (left) and CD8^+^ (right) lymphocyte subpopulations concentration at week 14 (values are shown in cells per milliliter) depend on clinical remission. Similar results were found between patients with biochemical or endoscopic improvement and patients without improvement.
