# Supplementary figures and images for: Memory T Cell Subpopulations as Early Predictors of Remission to Vedolizumab in Ulcerative Colitis
Source: Front Med (Lausanne). 2022 Jun 15;9:837294. doi: 10.3389/fmed.2022.837294 (PMC9240758; doi:10.3389/fmed.2022.837294)

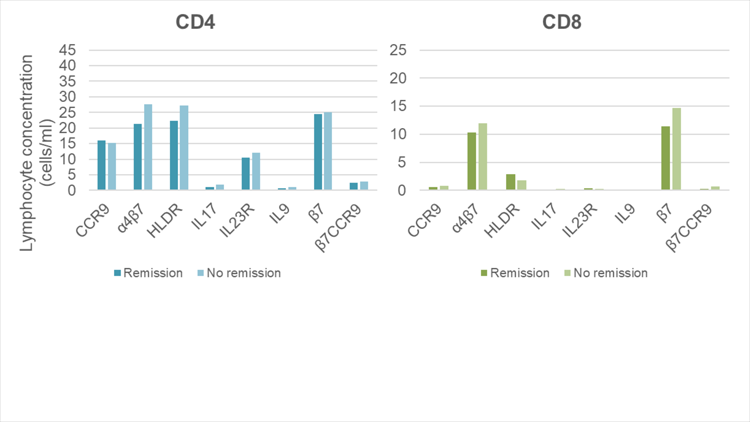

Supplement: Supplementary Figure 2 — Median of CD4+ (left) and CD8+ (right) lymphocyte subpopulations concentration at week 6 (values are shown in cells per milliliter) depend on clinical remission. Similar results were found between patients with biochemical or endoscopic improvement and patients without improvement. [file Image_2.TIF]

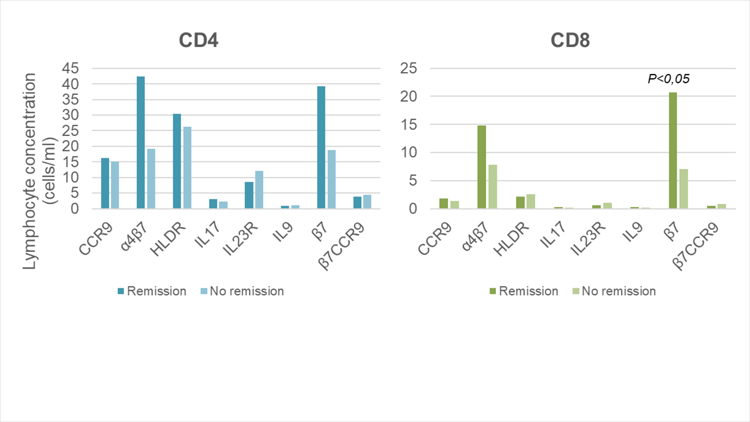

Supplement: Supplementary Figure 3 — Median of CD4+ (left) and CD8+ (right) lymphocyte subpopulations concentration at week 14 (values are shown in cells per milliliter) depend on clinical remission. Similar results were found between patients with biochemical or endoscopic improvement and patients without improvement. [file Image_3.TIF]
